# Pancreatic Insulinoma Masquerading as Neurological Disease: Diagnosis and Treatment of a Rare Tumor

**DOI:** 10.7759/cureus.61378

**Published:** 2024-05-30

**Authors:** Seun A Arowolo, Muzamil Khan, Eaman Hamid, Nusiba Ahmed, Eithar Shabbo, Hira Nasir

**Affiliations:** 1 Internal Medicine, Obafemi Awolowo University, Ife, NGA; 2 Internal Medicine, George Washington University School of Medicine and Health Sciences, Washington D.C., USA; 3 Medicine, Omdurman Islamic University, Omdurman, SDN; 4 Medicine, Alzaiem Alazhari University, Khartoum, SDN; 5 Medicine, Ahfad University for Women, Khartoum, SDN; 6 Internal Medicine, Mayo Hospital, Lahore, PAK

**Keywords:** hyperinsulinemia, multiple endocrine neoplasias, neuroglypenia, hyperinsulinemic hypoglycemia, pancreatic insulinoma

## Abstract

Insulinomas are rare functional pancreatic neuroendocrine tumors that typically manifest with classic hypoglycemic symptoms, such as diaphoresis, palpitations, and tremors. Although infrequent, neuroglycopenic symptoms associated with insulinomas have been reported, often leading to delayed diagnoses. Here, we present the case of a 31-year-old male with pancreatic insulinoma who experienced recurrent episodes of seizures and confusion preceded by diaphoresis, tremors, and palpitations. During these episodes, he was found to be hypoglycemic. Comprehensive evaluations, including brain and abdominal imaging, as well as biochemical and serological testing, were conducted. The findings confirmed a diagnosis of pancreatic insulinoma. The patient underwent surgical resection of the tumor, and a biopsy confirmed the insulinoma diagnosis. He remained asymptomatic during subsequent follow-ups.

## Introduction

Insulinomas are rare but the most common functional type of pancreatic neuroendocrine tumor, with a global incidence of four cases per one million person-years [1]. Most cases of insulinoma are benign, and malignant cases are mainly associated with larger sizes and associations with other polyendocrine tumors. Patients with symptomatic insulinoma may present with classic symptoms of hypoglycemia, which include diaphoresis, tremors, palpitations, or confusion. These symptoms are mainly due to endogenous hyperinsulinemia [2]. Although rare, patients with insulinoma may also present with neurological symptoms, including seizures, confusion, limb weakness, peripheral neuropathy, behavioral changes, or coma [3]. We present a case of pancreatic insulinoma presenting primarily with neurologic manifestations, emphasizing the challenges in the diagnosis and management of this rare condition.

## Case presentation

A 31-year-old male with no previous medical history was brought to the emergency department with recurrent episodes of confusion and two episodes of generalized tonic-clonic seizures in the last four weeks. Each episode of confusion was preceded by diaphoresis, palpitations, and tremors and resolved promptly after sugary food ingestion. His family also complained that he had experienced unexplained episodes of loss of consciousness, appeared well in between episodes, and had been increasingly irritable. He had no history of alcohol, smoking, or substance abuse. He also reported no history of family disease.

**Figure 1:**
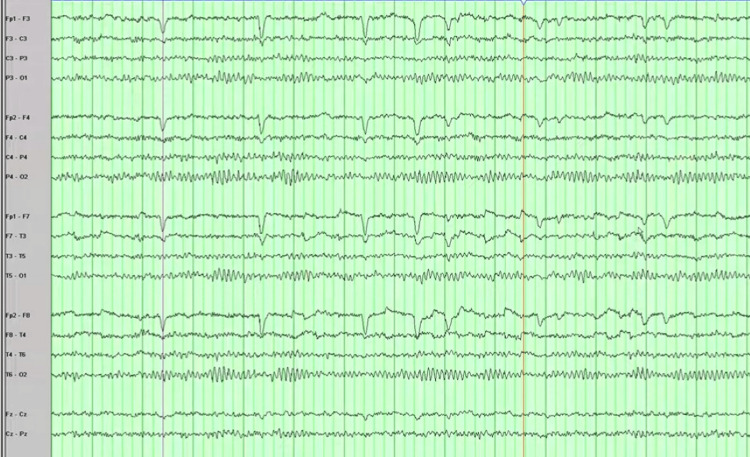
Normal encephalogram with most waves of 8 Hz and higher frequencies.

On examination, he was anxious, diaphoretic, afebrile, and hemodynamically stable. He was well-oriented to time, place, and person, with no neurological deficit. There were no signs of meningeal irritation, and cranial nerves were intact. He underwent brain computed tomography (CT) without contrast, which was unremarkable, and an electroencephalogram (EEG) revealed no abnormality (Figure 1). His cerebrospinal fluid analysis is shown in Table 1. His initial laboratory evaluations were within the normal range except for hypoglycemia (57/mg/dl, normal: 70-140). He was started on valproic acid after a probable diagnosis of epilepsy was made. On discharge day, he again experienced generalized tonic-clonic seizures, preceded by diaphoresis, tachycardia, palpitations, and tremors, and became confused. He was found to be hypoglycemic during the episode, and his condition improved after intravenous dextrose saline. Valproic acid was discontinued, and levetiracetam was initiated.

**Table 1:** Cerebrospinal fluid analysis.

Parameter	Lab value	Reference range
Appearance	Clear	Clear
Pressure	12	5-20 cmH_2_0
Protein	41	15-45 mg/dl
Glucose	3.1	2.5-3.5
White cell count	2	< 05 cells
Gram stain	Negative	Negative
Red cell count	1	< 10 cells

Due to recurrent hypoglycemia, an endocrinologist consultation was made. On questioning, he reported a five-year history of tachycardia, dizziness, and other symptoms that used to vanish immediately after candy ingestion. He underwent a series of biochemical tests, and the results are shown in Table 2.

**Table 2:** Lab results during admission. BHB: beta-hydroxybutyrate

Parameter	Lab result	Reference value	Lab result for insulinoma suspicion
Blood glucose	39 mg/dl	70-100	≤ 40
Insulin	3.34 µU/ml	< 0.025	≥ 3
Proinsulin	4.44 pmol/L	< 0.022	≥ 5
C-peptide	0.85 ng/L	0.2-0.8	≥ 0.2
BHB	0.11 mmol/L	< 0.5	≤ 2.7
Other hypoglycemic agents in serum	No	No	No

**Figure 2:**
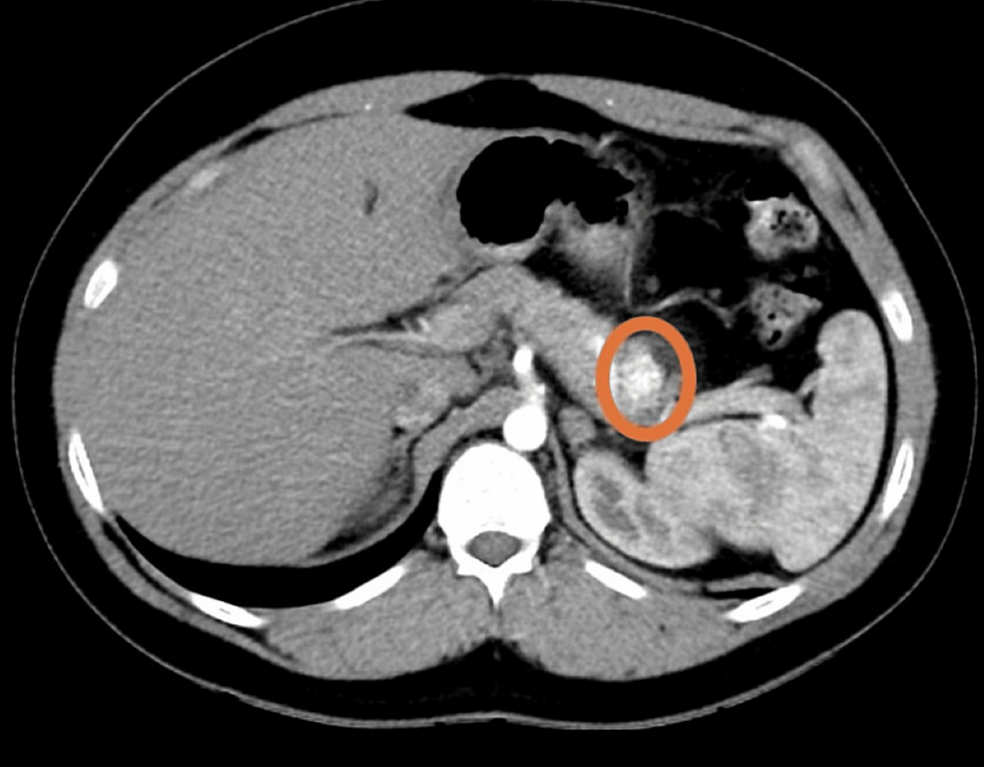
Abdominal computed tomography revealing well-defined hyperdense lesion in the body pancreas.

**Figure 3:**
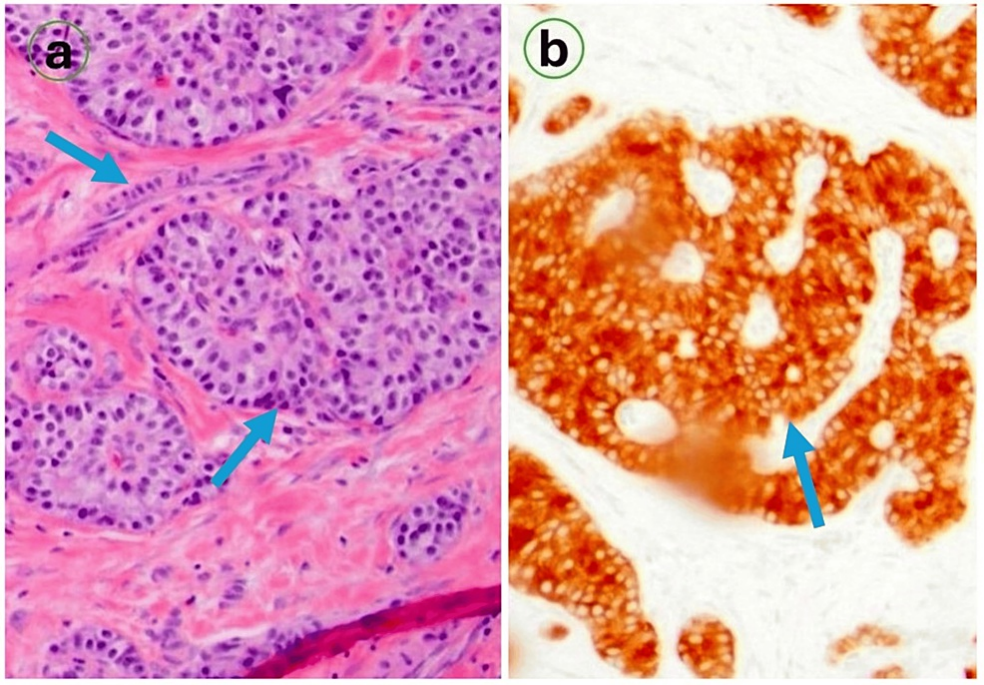
Histopathology findings of biopsy sample: nests of polygonal cells with abundant eosinophilic cytoplasm (a), tumor cell staining positive for insulin (b). Stains: eosin, hematoxylin; image (a): magnification x100; image (b): magnification x400

Abdominal contrast-enhanced CT revealed a heterogenous mass in the pancreatic tail, with suspicion of a pancreatic tumor with no distant metastasis (Figure 2). Based on his clinical picture, serological findings, and imaging results, a probable diagnosis of insulinoma was made. Consultation with a general surgeon was advised, who recommended a distal pancreatectomy. After receiving informed consent, he underwent surgical removal of the distal pancreas with a tumor. He remained hospitalized for the next three days, remained euglycemic, and developed no neurological manifestations. The surgical pathology of the lesion revealed a well-differentiated neuroendocrine tumor, and staining was positive for insulin and synaptophysin, consistent with insulinoma (Figure 3).

Two weeks later, at follow-up, he was screened for multiple endocrine neoplasia type 1 (MEN-1); however, no evidence of pituitary or parathyroid dysfunction was noted. At his six-month follow-up, he remained asymptomatic, and his glucose level (fasting and post-prandial) was within the normal range.

## Discussion

Insulinoma is a rare neuroendocrine tumor arising from the pancreatic beta cells. Insulinomas are primarily benign, and malignant cases are rare, mainly associated with larger sizes and elevated insulin and proinsulin levels [4]. Insulinoma can occur at any age, in both males and females [5]. The exact etiology of insulinoma remains unknown, and sporadic cases are more common than familial ones. Familial cases of insulinoma have an association with MEN-1 syndrome, characterized by a predisposition to multiple endocrine tumors, including insulinoma, pituitary, and parathyroid adenoma [5]. However, most cases are sporadic and arise spontaneously, without any genetic predisposition.

Clinical manifestations of insulinoma are hypoglycemia, neuroglycopenia, and the release of catecholamines in response to hypoglycemia (Table 3) [6].

**Table 3:** Reported manifestations of insulinoma. [6]

Symptoms	Number of patients	Percentage (%)
Weakness	59	88
Palpitations	57	85
Sweating	58	87
Coma	49	73
Seizures	33	50
Behavioral problems	47	70

Insulinomas typically present with clinical symptoms related to hypoglycemia. The classic triad of symptoms is known as Whipple’s triad (Table 4) [7]. In some cases, insulinomas may present with neuroglycopenic and adrenergic symptoms and may lead to misdiagnosis initially. We have tabulated the diagnostic criteria for insulinoma in Table 4 [8].

**Table 4:** Diagnostic criteria for insulinoma. BHB: beta-hydroxybutyrate [8]

Clinical criteria
Hypoglycemic symptoms
Hypoglycemia (plasma glucose < 50 mg/dl)
Prompt relief of symptoms after administration of glucose
Lab diagnostic criteria
Insulin ≥ 3 µU/ml
Proinsulin ≥ 5 pmol/L
BHB ≤ 2.7 mmol/L
C-peptide ≥ 0.2 ng/dl mmol/L
Negative plasma sulfonylurea

Localization of insulinomas is done using the following modalities, including abdominal CT, endoscopic ultrasound, or magnetic resonance imaging with dynamic sequences [9]. The primary treatment for symptomatic insulinomas is surgical resection while preserving pancreatic function. Complete enucleation is often recommended due to its benign and solitary nature. Distal pancreatectomy or the Whipple procedure are alternatives to enucleation [10]. Medical management, including octreotide or diazoxide, may be considered in cases where surgery is contraindicated or there are unresectable tumors [11].

Our patient presented with adrenergic and neuroglycopenic symptoms. He was found to be hypoglycemic during episodes. His brain CT was normal. His lab revealed hypoglycemia and hyperinsulinemia. His abdominal CT revealed a well-defined, hyperdense lesion in the body of the pancreas. He was diagnosed with a probable diagnosis of insulinoma. He underwent a surgical resection of the tumor, and the biopsy specimen revealed a well-differentiated neuroendocrine tumor.

## Conclusions

Although rare, insulinoma is the most common cause of hypoglycemia in patients with endogenous hyperinsulinism. Due to their atypical clinical presentation, insulinomas are challenging to diagnose. Insulinomas should be included in the differential diagnoses of patients presenting with recurrent neuroglycopenic and adrenergic symptoms attributed to hypoglycemia. Timely diagnosis and localization of insulinomas are essential for appropriate surgical management and improved outcomes for the affected patients.
